# The ability to suppress macrophage-mediated inflammation in orbital fat stem cells is controlled by miR-671-5p

**DOI:** 10.1186/scrt486

**Published:** 2014-08-13

**Authors:** Gi-Shih Lien, Jen-Fang Liu, Ming-Hsien Chien, Wei-Tse Hsu, Tzu-Hao Chang, Chia-Chi Ku, Andrea Tung-Qian Ji, Peng Tan, Ting-Lieh Hsieh, Liang-Ming Lee, Jennifer H Ho

**Affiliations:** Department of Internal Medicine, Wan Fang Hospital, Taipei Medical University, 111 Hsing-Long Road, Sec. 3, Taipei, 116 Taiwan; School of Nutrition and Health Science, 250 Wu-Hsing Street, Taipei, 110 Taiwan; Research Center for Industry of Human Ecology, Chang Gung University of Science and Technology, 261 Wen-hwa 1st Road, Kwei-shan, Taoyuan 333 Taiwan; Center for Stem Cell Research, Wan Fang Hospital, Taipei Medical University, 111 Hsing-Long Road, Sec. 3, Taipei, 116 Taiwan; Graduate Institute of Clinical Medicine, Taipei Medical University, 250 Wu-Hsing Street, Taipei, 110 Taiwan; Graduate Institute of Biomedical Informatics, Taipei Medical University, 250 Wu-Hsing Street, Taipei, 110 Taiwan; Department of Urology, Wan fang Hospital, Taipei Medical University, 111 Hsing-Long Road, Sec. 3, Taipei, 116 Taiwan; Department of Ophthalmology, Wan Fang Hospital, Taipei Medical University, 111 Hsing-Long Road, Sec. 3, Taipei, 116 Taiwan

## Abstract

**Introduction:**

Our previous works demonstrated that systemic orbital fat-derived stem cell (OFSC) transplantation was effective in ameliorating lipopolysaccharide (LPS)-induced extensive acute lung injury (ALI) *in vivo* mainly through paracrine regulation of macrophage-mediated cytokine-storm. In this study, we explore the molecular mechanism(s) of OFSCs regulating macrophage activity in a cytokine-inducible fashion.

**Methods:**

LPS (100 ng/ml)-activated macrophages were treated by conditioned medium from OFSCs (OFSCs-CM) or non-contact cultured with OFSCs for 6 hours. The potency of OFSCs on macrophage proliferation and pro-inflammation ability were determined. Expression levels of pro-inflammatory cytokines in macrophages, inducible immuno-modulatory factors in OFSCs, were investigated. Deep sequencing analysis as well as interaction between microRNA (miRNA) and genes of immuno-modulators in OFSCs induced by activated macrophages was predicted by miRTar. Transfection of miRNA inhibitor into OFSCs was performed. Real-time RT-PCR and transplantation of OFSCs into mice with LPS-induced ALI confirmed the *in vitro* and *in vivo* mechanism.

**Results:**

The paracrine effect of OFSCs on inhibition of macrophage pro-inflammatory cytokine release was more potent than induction of macrophage G0/G1 cell cycle arrest. OFSCs-CM suppressed LPS-induced inducible nitric oxide synthetase and the pro-inflammatory cytokines such as tumor necrosis factor-alpha (TNF-α), interleukin (IL)-1 alpha, and IL-1 beta expression in macrophages. Under non-contact culture, LPS-activated macrophages effectively triggered the expression of soluble immuno-modulating factors in OFSCs, i.e., IL-10, IL-1 receptor antagonist (IL-1 RA), indoleamine 2,3-dioxygenase, and soluble TNF receptor type II (sTNF RII). Under miRTar prediction, miR-671-5p was identified as a critical microRNA in regulation of multiple immune-modulating factors in OFSCs response to macrophages. The baseline level of miR-671-5p was high in OFSCs, and down-regulation of miR-671-5p upon co-culture with activated macrophages was observed. MiR-671-5p inhibitor transfection into OFSCs selectively enhanced the IL-1 RA and sTNF RII expressions. In addition, inhibition of miR-671-5p in OFSCs enhanced the anti-inflammatory ability against LPS-induced ALI.

**Conclusion:**

The paracrine effect of OFSCs inhibits the pro-inflammatory ability and proliferation of macrophages. The immune-modulation capacity of OFSCs can be triggered by activated macrophages, and down-regulation of miR-671-5p enhances OFSC immuno-modulation ability by up-regulating IL-1 RA and sTNF RII expression.

## Introduction

Acute respiratory distress syndrome accounts for the major mortality of acute lung inflammation [1], which can be triggered by various pathogens including atypical infection; that is, severe acute respiratory syndrome. Cytokine storm-mediated extensive lung injury is the ultimate pathomechanism of acute respiratory distress syndrome and severe acute respiratory syndrome [2, 3]. In addition to specific antibiotics and antiviral agents, steroid treatment and plasma exchange are therapeutic strategies to reduce local and circulating inflammatory cytokine levels. There is no safe and effective therapy to eliminate cytokine storm in critical patients since severe steroid-related and plasmapheresis-associated complications may occur in severely ill patients [4, 5].

The mesenchymal stem cell (MSC) is the only stem cell with the capacity for allogeneic transplantation without matching human leukocyte antigen typing due to the low immunogenecity [6–8]. Except for differentiation ability [9], the MSC as an immunomodulator is a powerful therapeutic strategy in graft versus host disease, autoimmune neurological disease, systemic lupus nephritis, acute lung tissue injury and diabetes [6, 10]. MSCs achieve immunomodulation effects on both innate and adaptive immunities by secreting critical soluble factors and/or direct contact regulation of immune cells [6, 7], and procytokines such as interferon gamma (IFNγ), interleukin (IL)-1β or tumor necrosis factor alpha (TNFα) stimulate the immunomodulatory ability of MSCs [11, 12]. Transforming growth factor beta (TGFβ), hepatocyte growth factor, IL-10, indoleamine 2,3-dioxygenase (IDO) and prostaglandin E_2_ are thought to be inducible immunomodulating factors secreted from MSCs upon procytokine stimulation for targeting T cells, B cells and natural killer cells [13–16].

Little is known about the effect of MSCs on macrophages, critical players of the innate immune response involved in almost all immune-mediated diseases. Only a few studies report that MSCs derived from bone marrow or gingiva promote the generation of regulatory macrophages (M2) [17–20]. Interleukin-1 receptor antagonist (IL-1RA) produced by MSCs serves as a key factor for inhibiting macrophage-mediated inflammation in acute lung injury (ALI) [21]. Our previous work demonstrates that orbital fat-derived stem cells (OFSCs), MSCs isolated from human orbital fat tissues [22], are effective in modulating lipopolysaccharide (LPS)-induced acute lung inflammation [23]. The therapeutic effect of OFSCs *in vivo* is attributed to inhibition of macrophage-mediated inflammatory response, and the paracrine effect of OFSCs contributes the major therapeutic benefit. However, the circulation cytokine profile altered by OFSCs is not identical to that altered by bone marrow-derived MSCs in mice with ALI.

In this study, we investigate the molecular mechanism of OFSCs on macrophage regulation through paracrine effects. LPS-activated macrophages were treated with condition medium of OFSCs (OFSCs-CM) or were noncontact cultured with OFSCs. Changes in macrophages and OFSCs as well as the role of microRNAs (miRNAs) in OFSCs regulating the soluble immunomodulatory factors induced by cytokines were elucidated.

## Materials and methods

### Isolation and culture of orbital fat-derived stem cells

Isolation and culture of the OFSCs were performed as described previously [22]. All samples were removed with the written informed consent of the subjects and this study was approved by the Institutional Review Board of Taipei Medical University-Wan Fang Hospital. In brief, redundant orbital fat tissue was removed from the intraorbital cavity. Adipose tissues were fragmented, digested and filtered. After centrifuging the fluid, cells from the resulting pellet were plated in tissue culture flasks (BD Biosciences, Franklin Lakes, NJ, USA) and maintained in Mesen Pro Medium (Invitrogen, Carlsbad, CA, USA). Surface phenotypes of OFSCs were positive for MSC markers (CD29, CD90, CD105) and negative for hematopoietic markers (CD31, CD34, CD45, CD106), and the trilineage differentiation ability of these cells has been checked previously [22].

### Culture of macrophages

The mouse macrophage cell line RAW264.7 was obtained from the American Type Culture Collection (Livingstone, MT, USA), and cells were maintained in Dulbecco’s modified Eagle’s medium (DMEM)/Ham’s F-12 nutrient mixture containing 10% fetal calf serum, 100 U/ml penicillin G, and 100 μg/ml streptomycin in a humidified 37°C incubator.

### Macrophage–orbital fat-derived stem cell co-culture studies

Macrophages were incubated with serum-free medium (DMEM/Ham’s F12 medium) for 24-hour starvation before co-culture with OFSCs or treated OFSCs-CM. OFSCs were maintained in low serum medium (Iscove's Modified Dulbecco's Media (IMDM) containing 1% fetal bovine serum) for 24-hour starvation before co-culture with macrophages or condition medium collection. In this study, *Escherichia coli* O55:B5-produced LPS (Sigma-Aldrich, St Louis, MO, USA) was added to the co-culture system or OFSC-CM-treated macrophages at a final concentration of 10 to 1,000 ng/ml.

In a transwell culture system, macrophages (5 × 10^5^/well for protein analysis and flow cytometry, and 1 × 10^5^/well for other experiments in this study) with or without LPS stimulation were seeded into 0.4 μm pore inserts of transwells (BD Falcon, Franklin Lakes, NJ, USA) and co-cultured with various numbers of OFSCs. The ratio of OFSC numbers versus macrophage numbers in the transwell system was adjustable from 0.5 to 4. During the period of co-culture, cells were incubated in 1:1 mixed medium (one-half DMEM/Ham’s F12 medium for macrophage and one-half IMDM containing 1% fetal bovine serum for OFSCs). After co-culture for 6 hours, a series of experiments were performed on macrophages and OFSCs, respectively.

For condition medium collection, various numbers of OFSCs (OFSC/macrophage ratio from 0.5 to 4) were plated in six-well culture plates. The condition medium was collected from OFSCs under 24-hour starvation. Macrophages were then treated with 1:1 mixed medium (one-half DMEM/Ham’s F12 medium for macrophages and one-half OFSCs-CM). After 6 hours of OFSC-CM treatment, studies on functional analysis of macrophages were performed.

### Flow cytometric analysis

For cell cycle analysis, cells were harvested with cell scraper, washed twice and fixed in 70% ethanol at –20°C. Nuclear DNA was stained with propidium iodide (50 mg/ml). After blocking with Fc receptor for 15 minutes at 4°C, CD 206 or isotype-matched control antibody (BD Biosciences, San Jose, CA, USA) was added and incubated for 30 minutes at 4°C in the dark and was analyzed using FACSCalibur (Becton Dickinson, Franklin Lakes, NJ, USA) and FlowJo software (Tree Star, Ashland, OR, USA).

### Cell viability analysis

The macrophages were harvested and resuspended in phosphate-buffered saline. The cellular suspension was mixed with equal amounts of trypan blue solution and the number of live (transparent) and dead (blue) cells were counted using a hemacytometer (Assistent, Sondheim, Germany).

### Western blot analysis

The cell lysates were prepared as described previously [24]. Western blot analysis was performed using primary antibodies against inducible nitric oxide synthase (iNOS) (0.1 μg/ml), TNFα (0.1 μg/ml), IL-1β (0.1 μg/ml), TGFβ (0.125 μg/ml), CD14 (0.1 μg/ml), soluble tumor necrosis factor receptor (sTNFR) type II (2 μg/ml), IL-1RA (2.5 μg/ml) (Abcam, Cambridge, MA, USA), or CD68 (0.9 μg/ml; Epitomics, Burlingame, CA, USA). The density of protein bands was assessed using a computing densitometer with Image-Pro plus software (Media Cybernetics, Inc., Bethesda, MD, USA).

### Real-time quantitative reverse transcription polymerase chain reaction

Total RNAs from OFSCs and macrophages were isolated using Trizol (Invitrogen, Carlsbad, CA, USA). Reverse transcription was performed using MMLV reverse transcriptase (Invitrogen). One microliter of the reverse transcription product was amplified using primer pairs specific for miR-671-5p, IFNγ, TNFα, IL-1α, IL-1β, TGFβ, IL-10, IL-1RA, sTNFR type II, and IDO. GAPDH, 18S, and RUN44 were used as controls for quantitation. Real-time polymerase chain reactions were conducted in an ABI Prism 7300 Sequence Detection System using SYBR Green PCR core reagents (Applied Biosystems, Foster City, CA, USA). The forward and reverse primers for the amplifications are presented in Table 1. Primers for miR-671-5p were purchased from Ambion, and miR-671-5p levels were assayed following the manufacturer’s protocol.

**Table 1:** **Primer sequences used in this study**

Primers used in QPCR	Sequence (5′ to 3′)
Human 18S-F	ATGGCCGTTCTTAGTTGGTG
Human 18S-R	AACGCCACTTGTCCCTCTAA
Human IDO-F	CCCAAAGGAGTTTGCAGGGGGC
Human IDO-R	GCCCAGCAGGACGTCAAAGCA
Human IL-1RA-F	ACCTGCCCAACCTGCTCTCCT
Human IL-1RA-R	GGAGCCACTTGGTTGGGGGTCA
Human IL-10-F	TGTTGAGCTGTTTTCCCTGA
Human IL-10-R	TTGTAGCAGTTAGGAAGCCC
Human sTNFR-II-F	CCAGTCTTGTGTCTGCGTCT
Human sTNFR-II-R	GAGGGGGAAGGCTGACTCTA
Human TGFβ-F	GCAACAATTCCTGGCGATAC
Human TGFβ-R	CTAAGGCGAAAGCCCTCAAT
Mouse GAPDH-F	CCCCAGCAAGGACACTGAGCAAG
Mouse GAPDH-R	GGGGTCTGGGATGGAAATTGTGAGG
Mouse IFNγ-F	AACCCACAGGTCCAGCGCCA
Mouse IFNγ-R	CACCCCGAATCAGCAGCGACT
Mouse TNFα-F	CAACGCCCTCCTGGCCAACG
Mouse TNFα-R	TCGGGGCAGCCTTGTCCCTT
Mouse IL-1α-F	AGCTTGACGGCACCCTCGCA
Mouse IL-1α-R	CGGAGAGCTTCGTGGCTGTGGA
Mouse IL-1β-F	AACCGGCGCTGGAACTG
Mouse IL-1β-R	GGTCCCTTGTGTCACCACCTT

F, forward; IFNγ, interferon gamma; IDO, indoleamine 2,3-dioxygenase; IL, interleukin; IL-1RA, interleukin-1 receptor antagonist; QPCR, quantitative polymerase chain reaction; R, reverse; sTNFR, soluble tumor necrosis factor receptor; TGFβ, transforming growth factor beta; TNFα, tumor necrosis factor alpha.

### Deep sequencing and microRNA analysis

Sequencing analysis was performed in OFSCs from three different donors using the Illumina Solexa sequencer, and a total read of 25,068,914 was generated from each sample. The FASTX-Toolkit [25] was used to trim adapter sequences and remove low-quality reads. miRDeep2 [26] was used to identify miRNAs, and the expression of miRNAs were normalized to obtain the expression of transcripts per million. A total of 439 miRNAs in miRBase [27] were detected in OFSCs, and 34 out of 439 miRNAs was selected as highly expressed miRNAs. miRTar [28] was applied to predict interactions between the 34 highly expressed miRNAs and genes involved in secreted immunomodulating factors in MSCs (*IL-6*, *IL-10*, *IDO*, *HGF*, *TGFβs*, *sTNFRI*, *sTNFRII*, and *IL-1RA*) or proinflammatory cytokines in macrophages (*TNF-α*, *IFN-γ*, *IL-1α*, *IL-1β*)*,* respectively. miRanda [29] was applied for analysis of miRNA–target interactions, and generated the minimal free energy and alignment score.

### Transfection of miR-671-5p

OFSCs were transfected with the miR-671-5p inhibitor (Dharmacon, Bonn, Germany) at a final concentration of 5 nM using GenMute™ siRNA Transfection Reagent (SignaGen Laboratories, Pittsburgh, PA, USA) according to the manufacturer’s instructions. The level of miR-671-5p in OFSCs with or without macrophage co-culture was measured by real-time reverse transcription polymerase chain reaction after 6 hours of miR-671-5p inhibitor transfection, and the putative target gene expressions in OFSCs were evaluated under co-culture experiments.

### Animal experiments

Male Balb/c mice were maintained in the animal facility and all experimental protocols were approved by the animal use and care committee of Taipei Medical University-Wan Fang Hospital. The animal model of LPS-induced ALI was established as per our previous report [23]. Briefly, LPS (25 μg in 50 μl sterile saline/mice) was delivered into 8-week-old to 10-week-old mice (BioLASCO Taiwan Co., Ltd, Taipei, Taiwan) via intratracheal injection. Twenty minutes after LPS injection, 3 × 10^5^ OFSCs with or without transfection of miR-671-5p inhibitor in 50 μl phosphate-buffered saline were administrated via the tail vein. Tail vein injection of 50 μl phosphate-buffered saline served as the control. Animals were sacrificed 6 hours after LPS exposure. Lung tissues were removed and stained with hematoxylin and eosin. Each condition was repeated in at least three independent mice.

### Detection of malondialdehyde level

The malondialdehyde level in lung tissue was detected by the Lipid Peroxidation (MDA) Assay Kit (Abcam). Tissue (10 mg) was homogenized on ice in 300 μl of the MDA Lysis Buffer (Abcam) and then centrifuged at 13,000 × *g* for 10 minutes to remove insoluble materials. Then 200 μl of the supernatant and 600 μl TBA solution were incubated at 95°C for 60 minutes before cooling down to room temperature in the ice bath for 10 minutes. The absorbance at 532 nm was read and proportioned to the malondialdehyde level.

### Statistical analysis

Values are shown as the mean ± standard error of the mean. Statistical analysis was performed using the Statistical Package for Social Science software, version 16 (SPSS Inc., Chicago, IL, USA). Data comparisons were performed with the Student’s *t* test when two groups were compared at *P* < 0.05. One-way analysis of variance analysis followed by Tukey’s *post hoc* test was used when more than three groups were analyzed. Different characteristics represented different levels of significance. Differences were considered significant at the 95% confidence interval.

## Results

### Orbital fat-derived stem cells inhibit LPS-induced macrophage activation

**Figure 1:**
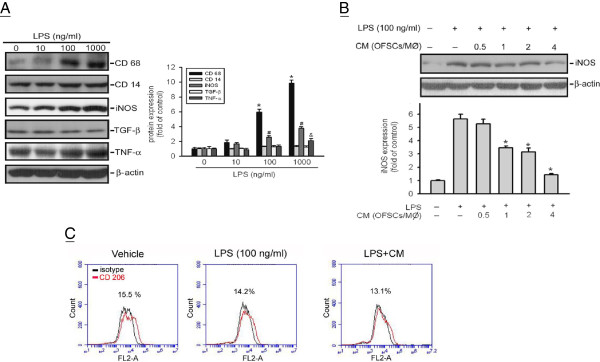
**Inhibition of inducible nitric oxide synthase production from macrophages by the paracrine effect of orbital fat-derived stem cells. (A)** Lipopolysaccharide (LPS) dose-dependently enhanced CD68 expression and triggered inducible nitric oxide synthase (iNOS) and tumor necrosis factor alpha (TNFα) production. **(B)** Condition medium of orbital fat-derived stem cells (OFSCs-CM) inhibited LPS-induced iNOS production at a ratio of OFSCs/macrophages (MØ) of 1 and higher. **(C)** Neither LPS nor OFSCs-CM altered CD206 expression on MØ. *t* test, *P < 0.05, *n* = 3. TGFβ, transforming growth factor beta.

In our previous study, LPS triggered CD68-expressing macrophage infiltration and OFSCs inhibited activation of the toll-like receptor 4/CD14/iNOS pathway in lung parenchyma [23]. In the present study, macrophages were treated with LPS at various concentrations (10 to 1,000 ng/ml) for 6 hours. LPS dose-dependently increased the expression of CD68, iNOS, and TNFα in macrophages (Figure 1A, left panel), and 100 ng/ml LPS and above significantly triggered the CD68 expression and iNOS production in macrophages (Figure 1A, right panel). A LPS concentration of 100 ng/ml was thus chosen for further experiments.

To determine the paracrine potency of OFSCs inhibiting iNOS production from LPS-activated macrophages, macrophages were treated for 6 hours with condition medium collected from various numbers of OFSCs depending on the ratio of OFSCs versus macrophages (OFSC/macrophage ratio from 0.5 to 4). OFSCs-CM dose-dependently decreased the iNOS production in macrophages induced by LPS (Figure 1B, left panel). OFSC/macrophage ratio up to 1 and higher significantly inhibited LPS-triggered iNOS production in macrophages (Figure 1B). However, neither 100 ng/ml LPS (Figure 1C, middle) nor OFSC-CM from OFSC/macrophage ratio of 1 (Figure 1C, right) altered CD206, a well-known marker for the M2 phenotype [30], on macrophages in the first 6 hours (Figure 1C, left).

### Orbital fat-derived stem cells induce cell cycle arrest on macrophages

**Figure 2:**
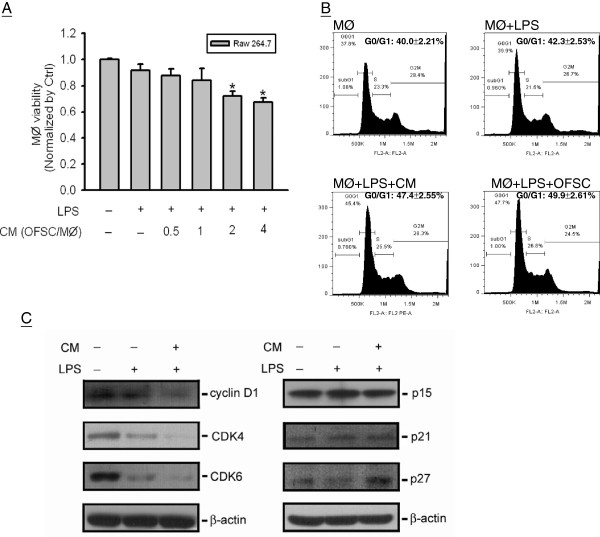
**Macrophage cell cycle arrest induction by orbital fat-derived stem cells. (A)** Condition medium of orbital fat-derived stem cells (OFSCs-CM) reduced the viability of macrophages (MØ) at OFSC/MØ ratio of 2 and higher. **(B)** Both OFSCs-CM and co-culture with OFSCs increased the G0/G1 population of macrophages. **(C)** OFSCs-CM reduced cyclin D_1_, CDK4, and CDK6 and increased p21^cip1^ and p27^kip1^ in MØ. *t* test, *P < 0.05, *n* = 3. LPS, lipopolysaccharide.

To study the paracrine effect of OFSCs on macrophage proliferation, numbers of macrophages were counted before and after treatment of OFSCs-CM with various concentrations for 6 hours. OFSC/macrophage ratio of 2 and higher significantly decreased macrophage numbers under LPS stimulation (Figure 2A). Flow cytometry on cell cycle analysis demonstrated that both OFSCs-CM and noncontact culture with OFSCs increased the G0/G1 population of macrophages (Figure 2B). Quantitative analysis for regulators of G1/S transition by western blot revealed that G1/S promoting factors such as cyclin D_1_, CDK4, and CDK6 in macrophages were reduced, while two CDK inhibitors (p21^cip1^ and p27^kip1^) were activated by OFSCs-CM (Figure 2C).

### Orbital fat-derived stem cells paracrine attenuate proinflammatory ability of macrophages

**Figure 3:**
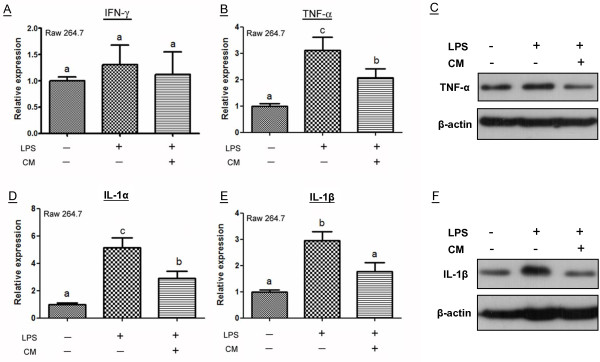
**Paracrine effect of orbital fat-derived stem cells attenuating proinflammatory ability of macrophages. (A)** Neither lipopolysaccharide (LPS) nor condition medium (CM) of orbital fat-derived stem cells (OFSCs) altered interferon gamma (IFNγ) expression in macrophages. OFSCs-CM inhibited LPS-induced tumor necrosis factor alpha (TNFα) **(B)**, interleukin (IL)-1α **(D)** and IL-1β **(E)** expression in macrophages. Analysis of variance with Tukey’s *post hoc* test, different characteristics represented different level of significance at 95% confidence interval, *n* = 3. OFSCs-CM reduced LPS-induced TNFα **(C)** and IL-1β **(F)** production in macrophages.

To evaluate the effect of OFSCs on proinflammatory capacity of macrophages, macrophages were first stimulated with LPS, while IFNγ, TNFα, IL-1α, and IL-1β served as the parameters of proinflammatory cytokines produced by LPS-activated macrophages. As shown in Figure 3, neither LPS nor OFSCs-CM altered IFNγ expression in macrophages (Figure 3A). OFSCs-CM effectively inhibited the proinflammatory ability of macrophages in the first 6 hours by reducing TNFα (Figure 3B), IL-1α (Figure 3D), and IL-1β (Figure 3E) expressions triggered by LPS. Twenty-four hours later, the protein levels of TNFα (Figure 3C) and IL-1β (Figure 3F) in macrophages were also reduced by OFSCs-CM.

### Lipopolysaccharide-activated macrophages trigger the immunomodulation capacity of OFSCs

**Figure 4:**
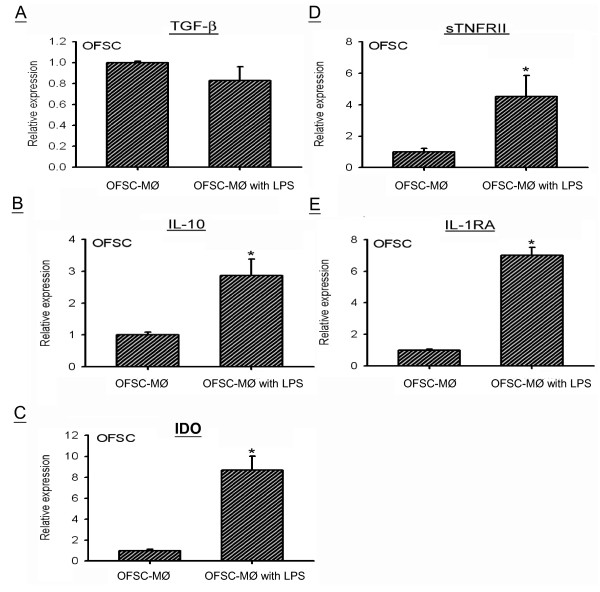
**Immunomodulatory excitation of orbital fat-derived stem cells by activated macrophages.** Upon noncontact culture, the lipopolysaccharide (LPS)-activated macrophages (MØ) did not alter transforming growth factor beta (TGFβ) expression **(A)**, but significantly upregulated interleukin (IL)-10 **(B)**, indoleamine 2,3-dioxygenase (IDO) **(C)**, soluble tumor necrosis factor receptor type II (sTNFRII) **(D)** and IL-1 receptor antagonist (IL-1RA) **(E)** expression in orbital fat-derived stem cells (OFSCs). *t* test, *P < 0.05, *n* = 3.

TGFβ, IL-10, IDO and IL-1RA are known as MSC-secreted factors in regulating innate immunity response to procytokines [12, 14, 15, 21]. Our previous *in vivo* study demonstrated that systemic OFSC transplantation enhanced the serum level of sTNFR type II in mice with LPS-induced ALI [23]. In the present study, OFSCs did express these immunomodulating factors when co-cultured with naïve macrophages (Figure 4). Furthermore, gene expression of IL-10 (Figure 4B), IDO (Figure 4C), sTNFR type II (Figure 4D) and IL-1RA (Figure 4E) in OFSCs were highly upregulated by LPS-activated macrophages within 6 hours of noncontact culture. TGFβ was not affected upon co-culture (Figure 4A).

### miR-671-5p expression in OFSCs responds to macrophage activation

Figure 4 shows that the mRNAs of IL-10, IDO, sTNFR type II and IL-1RA in OFSCs were rapidly increased by LPS-activated macrophages in a noncontact manner. Since a net mRNA expression may be affected by the dynamic balance of transcriptional regulation and post-transcriptional silencing mainly through miRNAs, deep-sequencing analysis and miRTar prediction were performed to identify the potential endogenous miRNAs in OFSCs, MSCs derived from subcutaneous tissue as well as MSCs derived from bone marrow targeting on inducible, secreted immunomodulating factors reported in MSCs (that is, IL-6, IL-10, IDO, hepatocyte growth factor, TGFβ, sTNFR type I, sTNFR type II, and IL-1RA). Seven miRNAs were found to potentially regulate the above genes by target prediction (Table 2). For OFSCs, the expression levels of hsa-miR-28-5p, hsa-miR-503 and hsa-miR-769-5p (transcripts per million < 1,000) were too low to act as a regulator for immunomodulation. hsa-let-7c, hsa-miR-370, and hsa-miR-423-5p were strongly expressed in OFSCs (transcripts per million > 2,000) but each of them targeted only one gene, making them less possible as the key regulator in this study. Hsa-miR-671-5p had a strong expression level (transcripts per million = 5,140) in OFSCs and potentially regulated those genes upregulated in LPS-activated macrophages, including IL-10, sTNFR type II and IL-1RA (Figure 4), indicating that miR-671-5p may be the key miRNA in OFSCs regulating inducible immunomodulating factors under procytokine stimulation.

**Table 2:** **Levels of microRNAs and their predicted target genes in OFSCs, ADSCs, and BMMSCs**

microRNA	Levels in OFSCs (TPM)	Levels in ADSCs (TPM)	Levels in BMMSCs (TPM)	Target genes
hsa-let-7c	11,978	4,119	994	sTNFR II
hsa-miR-28-5p	679	938	176	sTNFR II
hsa-miR-370	4,244	1,242	36	IL-6
hsa-miR-423-5p	2,661	2,566	219	sTNFR II
hsa-miR-503	716	1,570	208	IL-1RA
hsa-miR-769-5p	713	265	75	IL-10
hsa-miR-671-5p	5,140	1,253	18	IL-10, IL-1RA, sTNFR II

ADSC, mesenchymal stem cell derived from subcutaneous fat tissue; BMMSC, mesenchymal stem cell derived from bone marrow; IL, interleukin; IL-1RA, interlukin-1 receptor agonist; OFSC, orbital fat-derived stem cell; sTNFR II, soluble tumor necrosis factor receptor type II; TPM, transcripts per million.

### miR-671-5p regulates OFSC immunomodulation ability by targeting sTNFR type II and IL-1RA

**Figure 5:**
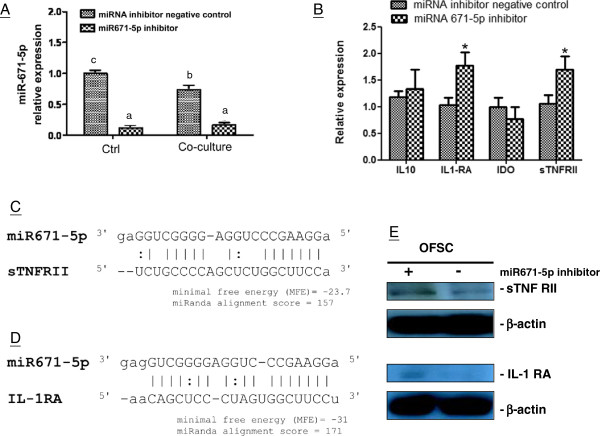
**MiR-671-5p targeting on soluble tumor necrosis factor receptor type II and interlukin-1 receptor agonist in orbital fat-derived stem cells. (A)** Co-culture with activated macrophages significantly reduced miR-671-5p in orbital fat-derived stem cells (OFSCs), and transfection of miR-671-5p inhibitor successfully inhibited miR-671-5p expression in OFSCs. Analysis of variance with Tukey’s *post hoc* test, different characteristics represented a different level of significance at 95% confidence interval, *n* = 3. **(B)** Transfection of miR-671-5p selectively upregulated interlukin-1 receptor agonist **(**IL-1RA) and soluble tumor necrosis factor receptor type II (sTNFRII) expression in OFSCs. *t* test, *P < 0.05, *n* = 3. miR-671-5p showed a strong binding affinity to sTNF RII **(C)** and IL-1RA **(D)** by a low minimal free energy and a high miRanda alignment score. **(E)** Inhibition of miR-671-5p enhanced the protein expressions of sTNFRII and IL-1RA in OFSCs. IDO, indoleamine 2,3 dioxygenase; IL, interleukin.

To determine whether miR-671-5p in OFSCs response to activated macrophages, we compared the level of miR-671-5p in OFSCs under normal conditions and noncontact cultured with LPS-activated macrophages. We showed that miR-671-5p expression was significantly downregulated in OFSCs when co-cultured with activated macrophages (Figure 5A). Furthermore, we identified the direct targets of miR-671-5p by transfection of miR-671-5p inhibitor into OFSCs. As shown in Figure 5A, miR-671-5p inhibitor successfully reduced the miR-671-5p expression in OFSCs both under normal conditions and co-culture with activated macrophages. Inhibition of miR-671-5p in OFSCs resulted in increasing the mRNA level of sTNFR type II and IL-1RA, but not IDO and IL-10 (Figure 5B). Notably, the binding sequences of miR-671-5p to sTNFR type II (Figure 5C) and IL-1RA (Figure 5D) demonstrated that the binding affinities between miR-671-5p and these two targets were strong, which is evidenced by a low minimal free energy and a high miRanda alignment score (Figure 5C,D). OFSCs expressed the low protein level of sTNFR type II and no IL-1RA protein could be detectable in OFSCs (Figure 5E). However, protein levels of both sTNFR type II and IL-1RA in OFSCs were increased after inhibiting miR-671-5p, which confirmed that cytokine levels in response to miR-671-5p were in line with the mRNA expression values (Figure 5E). The above findings confirmed that sTNFR type II and IL-1RA were direct targets of miR-671-5p.

### Inhibition of miR-671-5p enhances OFSCs anti-inflammation ability *in vivo*

**Figure 6:**
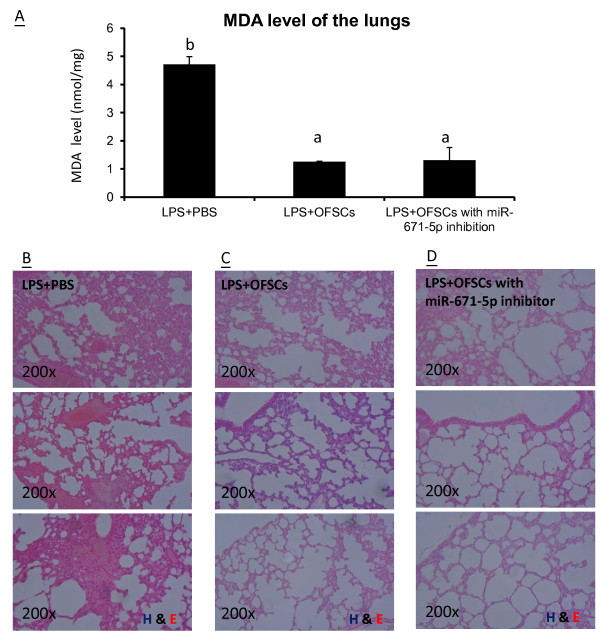
**Downregulation of miR-671-5p enhancing the** ***in vivo*** **anti-inflammation ability of orbital fat-derived stem cells. (A)** Systemic transplantation of orbital fat-derived stem cells (OFSCs) or OFSCs with miR-671-5p inhibitor significantly reduced the malondialdehyde (MDA) level within 6 hours in the lung tissues damaged by lipopolysaccharide (LPS). ANOVA with Tukey’s post hoc test, different characteristics represented different level of significance at 95% confidence interval, n=3. **(B)** LPS triggered severe inflammation and increased permeability in lung parenchyma in the first 6 hours. **(C)** Systemic OFSC transplantation ameliorated LPS-induced immune cells infiltration into lung parenchyma. **(D)** Inhibition of miR-671-5p enhanced the anti-inflammation ability of OFSCs on LPS-induced acute lung inflammation. H & E, hematoxylin and eosin; PBS, phosphate-buffered saline. (B) to (D), *n* = 3.

Finally, OFSCs with or without miR-671-5p inhibitor transfection were systemically injected into mice with LPS-induced ALI, and the therapeutic effect of both in the first 6 hours was compared. We measured the malondialdehyde level, a readout of lipid peroxidation, as the indicator of redox status in the lungs. The results showed that the redox status in a LPS-damaged lung parenchyma was significantly reduced by both OFSCs and OFSCs with preinhibition of miR-671-5p (Figure 6A). Tissue sections showed that LPS triggered the inflammatory cell infiltration into interstitial space of lung parenchyma, and increased lung permeability by fluid accumulation in alveolar space (Figure 6B). OFSC transplantation ameliorated lung permeability and inflammatory in the first 6 hours (Figure 6C). Moreover, less cell infiltration and larger alveolar space were noted in ALI mice receiving miR-671-5p-inhibited OFSCs compared with those mice receiving OFSCs (Figure 6D), demonstrating that preinhibition of miR-671-5p in OFSCs further enhanced the anti-inflammation ability in lung parenchyma without altering the antioxidative ability in OFSCs (Figure 6A).

## Discussion

**Figure 7:**
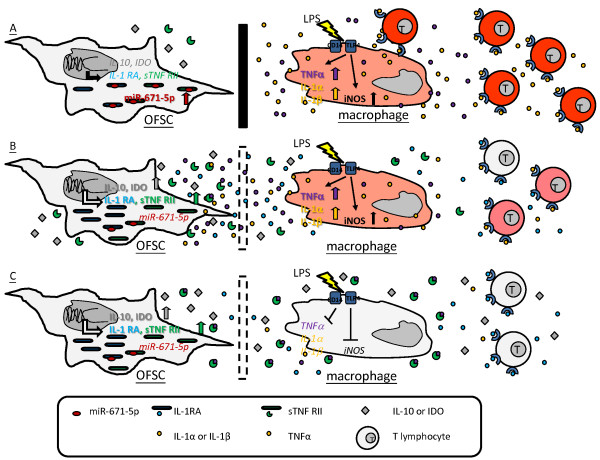
**Proposed schematic for miR-671-5p in orbital fat-derived stem cells regulating macrophage-mediated inflammation. (A)** Under normal conditions, soluble tumor necrosis factor receptor type II (sTNF RII) and interleukin-1 receptor antagonist (IL-1 RA) in orbital fat-derived stem cells (OFSCs) are negatively regulated by miR-671-5p, while lipopolysaccharide (LPS) stimulation promotes tumor necrosis factor alpha (TNFα), interleukin (IL)-1α and IL-1β expression in macrophages. **(B)** Upon noncontact culture with LPS-activated macrophages, IL-10 and indoleamine 2,3 dioxygenase (IDO) in OFSCs are upregulated, while sTNFRII and IL-1 RA are rapidly produced by degradation of miR-671-5p in OFSCs. **(C)** Abundant sTNF RII and IL-1 RA in OFSCs neutralizes the proinflammatory effect from TNFα, IL-1α and IL-1β. iNOS, inducible nitric oxide synthase; TLR4, toll-like receptor 4.

We first report that endogenous miR-671-5p participates in immunomodulation of MSCs by directly targeting sTNFR type II and IL-1RA, which are two inhibitory molecules. In this study, we find that LPS triggers the proinflammatory ability of macrophages (Figures 1, 3 and 7A), while OFSCs inhibit the proinflammatory activities (Figures 1, 3 and 7B) and induce cell cycle arrest (Figure 2) in macrophages through the paracrine effect. LPS-activated macrophages excite the immunomodulatory capacity of OFSCs via upregulation of IDO, IL-10, sTNFR type II and IL-1RA (Figures 4 and 7B,C). Among these factors, sTNFR type II and IL-1RA in OFSCs are negatively regulated by miR-671-5p under normal conditions (Figures 5 and 7A), and can be rapidly upregulated by degradation of miR-671-5p in OFSCs triggered by activated macrophages (Figures 4, 5 and 7B), which enhances the anti-inflammatory ability of OFSCs (Figures 6 and 7C).

According to our data, the condition medium from OFSCs significantly reduced the proliferation and proinflammatory ability in macrophages (Figures 1, 2 and 3), and the immunomodulatory ability of OFSCs could be induced by noncontact culture with LPS-activated macrophages (Figure 4), indicating that paracrine effects of OFSCs play an important role in macrophage regulation. In addition, the therapeutic potency of OFSCs for attenuating macrophage-mediated inflammation was stronger (Figure 1B) than inducing macrophage cell cycle arrest (Figure 2A). It is known that the number ratio of MSCs versus immune cells is critical for the therapeutic effect of immunomodulation in MSCs regulating lymphocytes (MSC/lymphocyte ratio > 0.1) [6, 31, 32] or natural killer cells (MSC/natural killer cells from 0.1 to 1) [15, 33]. We demonstrated that a ratio of OFSCs versus macrophages ≥1 (Figures 1B and 3) initiates anti-inflammation, while a ratio ≥2 initiates induction of macrophage cell cycle arrest (Figure 2).

Macrophages may initiate immune reaction through releasing proinflammatory cytokines including TNFα and IL-1 [34]. Macrophages resident in tissues can be polarized as proinflammatory macrophages (M1) or M2 by the microenvironment [35, 36]. M1 exhibit proinflammatory activity to activate T-helper type 1 lymphocytes, while M2 promote T-helper type 2 responses via increasing phagocytic activities [34–36]. Different from bone marrow-derived MSCs and gingiva-derived MSCs, OFSCs did not promote M2 generation (Figure 1C) but did significantly reduce the proinflammatory function of M1 by inhibition of TNFα, IL-1α and IL-1β (Figure 3) in the first 6 hours, indicating that orbital fat-derived MSCs regulate macrophages by targeting M1 rather than M2. However, we cannot exclude the possibility of M2 polarization induction by OFSCs through direct cell–cell interaction or long-term paracrine stimulation. In addition, we did measure the miR-671-5p expression in MSCs derived from different tissue origins. The data showed that miR-671-5p is strongly expressed in OFSCs, is moderately expressed in MSCs derived from subcutaneous fat tissue, and is at a very low level in MSCs derived from bone marrow (Table 2), indicating that regulation of M1 by miR-671-5p may predominantly exist in adipose tissue-derived MSCs.

IDO secretion from MSCs participating in immune tolerance and anti-inflammation could be stimulated by proinflammatory cytokines such as IL-1, IFNγ and TNFα [11, 12, 15, 16, 31]. From our data, LPS-activated macrophages enhanced the expression of TNFα, IL-1α and IL-1β (Figures 3B,C,D), and IDO in OFSCs was responsible for LPS-activated macrophage (Figure 4C). In addition to IDO, IL-10, sTNFR type II, and IL-1RA in OFSCs were also induced by activated macrophages (Figure 4).

IL-10 is a strong inhibitor for proinflammatory cytokine and chemokine release from activated macrophages [37]. Németh and colleagues report that bone marrow-derived MSCs reduce mortality and improve organ function in experimental cecal ligation-induced sepsis directly through increasing IL-10 production from LPS-activated macrophages [38]. According to our data, LPS-activated macrophages could also upregulate IL-10 expression in OFSCs (Figure 4B), and enhancement of IL-10 may justify the effect of OFSCs on suppression of macrophage proinflammatory cytokine release.

IL-1RA is a naturally occurring cytokine that is responsive to the physiological IL-1 level for neutralizing the action of IL-1α and IL-1β [39]. MSCs release IL-1RA to antagonize IL-1α and to block TNFα release from activated macrophages [21]. TNFα and IL-1 are two critical cytokines initiating a series of inflammatory response *in vivo*[40], and IL-RA-expressing MSCs can modulate the inflammatory response in mice with bleomycin-induced lung inflammation and fibrosis [21]. In addition to IL-1RA, sTNFR type I and sTNFR type II are other inhibitory soluble molecules found in body fluid as well as tissues that reduce the toxic effects of TNFα in the body [41]. sTNFR type II is one of the proteolytic shedding soluble extracellular domains of the tumor necrosis factor receptors secreted primarily by mononuclear cells [42]. Administration of TNFα or LPS to human MSCs increases the concentration of sTNFRs in the medium, suggesting that soluble receptors may be part of a negative feedback mechanism to inhibit the biological effects of TNFα [43]. Yagi and colleagues report that the therapeutic effect of an intramuscular injection of human bone marrow-derived MSCs in endotoxemic animals is dependent on the secretion of sTNFR type I [43]. Our previous work demonstrated that systemic OFSC transplantation upregulated serum level of sTNFR type II, not sTNFR type I, in the first 6 hours after intratracheal injection of LPS [23]. In this study, we demonstrated that both IL-1RA and sTNFR type II expressions in OFSCs were consequences in response to IL-1 and TNFα secretion by LPS-activated macrophages (Figure 4D,E).

Recently, miRNAs have been identified as critical regulators of immune responses [44, 45], and LPS stimulation may alter the expression of miRNAs in macrophages [46–48]. MiR-155 is upregulated in LPS-activated macrophages in response to TNFα interrupting macrophage differentiation and toll-like receptor 4 transcription [47–49]. In contrast, miR-125b is downregulated in LPS-treated macrophages for inhibiting TNFα expression [48]. However, we disclosed that miR-671-5p in MSCs derived from orbital fat tissues played a role in regulation of IL-1RA and sTNFR type II. Although miR-671-5p was predicted to interact with IL-10, IL-1RA and sTNFR type II (Table 1), only sTNFR type II and IL-1RA were directly inhibited by miR-671-5p (Figure 5B,C,D). Reduction of miR-671-5p in OFSCs in a macrophage-mediated proinflammatory environment (Figure 5A) resulted in the upregulation of sTNFR type II and IL-1RA in OFSCs (Figures 4D,E and 5B), which contributed to the anti-inflammation ability of OFSCs *in vivo* (Figure 6D).

## Conclusion

OFSCs inhibit macrophage-mediated inflammation and induce macrophage cell cycle arrest by the paracrine effect. The activated macrophages trigger immunomodulating factor expressions in OFSCs such as IDO, IL-10, IL-1RA and sTNFR type II. Upregulation of IL-1RA and sTNFR type II in OFSCs in a cytokine-inducible fashion is initiated by degradation of miR-671-5p.

## Authors’ information

G-SL is an Associate Professor in the Department of Internal Medicine, Taipei Medical University and also an Attending at the Division of Gastroenterology, Wan Fang Hospital. J-FL is a Professor working on immunology at the School of Nutrition and Health Science, Taipei Medical University and the Research Center for Industry of Human Ecology, Chang Gung University of Science and Technology. M-HC is an Associate Professor at the Graduate Institute of Clinical Medicine, Taipei Medical University. W-TH, C-CK, AT-QJ, PT and T-LH are research assistants in the Center for Stem Cell Research, Wan Fang Hospital, Taipei Medical University. T-HC is an Assistant Professor at the Graduate Institute of Biomedical Informatics, Taipei Medical University. L-ML is an Associate Professor in the Department of Urology, Taipei Medical University and also the Chief of the Division of Urology, Wan Fang Hospital. JHH is the Director of the Center for Stem Cell Research, a Consultant Ophthalmologist in the Department of Ophthalmology, Wan Fang Hospital, Taipei Medical University, and also an Associate Professor in the Graduate Institute of Clinical Medicine, Taipei Medical University.

## Abbreviations

ALI: acute lung injury
DMEM: Dulbecco’s modified Eagle’s medium
IDO: indoleamine 2,3-dioxygenase
IFNγ: interferon gamma
IL-1RA: interleukin-1 receptor antagonist
IL: interleukin
iNOS: inducible nitric oxide synthase
LPS: lipopolysaccharide
M1: proinflammatory macrophage
M2: regulatory macrophage
MSC: mesenchymal stem cell
miRNA: microRNA
OFSC-CM: condition medium of orbital fat-derived stem cell
OFSC: orbital fat-derived stem cell
sTNFR: soluble tumor necrosis factor receptor
TGFβ: transforming growth factor beta
TNFα: tumor necrosis factor alpha.
